# Supplementary material for: The Dual Prey-Inactivation Strategy of Spiders—In-Depth Venomic Analysis of Cupiennius salei
Source: Toxins (Basel). 2019 Mar 19;11(3):167. doi: 10.3390/toxins11030167 (PMC6468893; doi:10.3390/toxins11030167)
Supplement: Supplementary file 1 [file toxins-11-00167-s001.zip › Supplementary Fig S1.1-S1.19/Supplementary Fig S1.16.pdf]

Supplementary Fig S1.16

CLUSTAL O(1.2.4) multiple sequence alignment: Thyroglobulin type-1 domain-like proteins

```
TT1LP_1_CUPSA            MKAFIVVILACVATTSFCWEYPGYPGVDCPTAREKMLEQKD--VQWMIPECRADRTFVDL
TT1LP_2_CUPSA            MKAFIVVILACVATTSFCWEYPGYPGVDCPMAREKMLEQKD--VQWMIPECRADRTFVDL
TT1LP_VIRFA              MKVFIISILACTLSASLCWEYPGYPGVDCPTARERMLAQKD--AQWMIPKCHDDGTYVDL
tr_A0A0K1D8J1_DOLFI      MKAVCS--VIVF----SLCLALALAKTDCEEHRERELKSNT--KVKLVPKCDANGDYETL
sp_P84032_PHONI          ----------------------ARPKSDCEKHRESTEKTGT--IMKLIPKCKENSDYEEL
tr_A0A2P6KRP6_NEPCL      MKCVLL--LLAV----IVTVAVARPQSDCEKHREQAEKIGT--IMKLIPKCKENGDYEEL
tr_J7FXP5_LATHE          MKFIAS-VLALS----AICLAGALAATDCQEHRERELKSNS--KVKLIPKCTEEGDYEAL
tr_A0A2L2Z2A8_PARTE      ---ILI--ATVA----LSCHAGVFAKTACAEHREREQKSNT--KVKLAPKCTPEGDYEAL
tr_B5M6G6_HAPSC          MEGTFATIFLAVVAAVLCWEFPGHPGTDCPTTLKIMSEDPE--HRWMLPKCEEDGTFFPM
tr_A0A1D0C023_HADIN      --MYQFLIIVIL----AAFVNGAQEKTECQQHRERETRSRAPLPLRLIPECDENGEYKPL
tr_A0A224X890_MEGGE      ----MKLLVILS----IVYVVYAREKSECEVHREREQKNTA--LGRLIPECEENGDYKGL
tr_A0A1W7R943_HADSP      MKLFIL-VCFML----VLVTLSLAEQTPCQEKREKILSQNL--DVEVIPECEENGSYKDK
tr_T1DEL1_LYCBU          MKIYLTLGVFLV----LVAVYSAQEKSECEEHKEKAQKSSS--PVKVVPNCESNGDYAGL
tr_A0A1E1WVQ1_TITOB      MKKYLIFFIGLV----IITVSSAQKKSECQEHKEKAEKSTS--PVKVVPVCESNGDYAAL
                                                     *    :            : * *  :  :

TT1LP_1_CUPSA            QCYEKYPDVCMCVAPDGSPLTLPGFGVDPVTCVCFMAQYNIF---VHDPE-AEMPSCAKD
TT1LP_2_CUPSA            QCYEKYPDVCMCVAPDGSPLTLPGFGVDPVTCVCFMAQYNIF---VHDPE-AEMPSCAKD
TT1LP_VIRFA              QCYEKSPSTCMCVTPDGSPLTLPGFGLDVTTCVCFMVQYNIY---EHNPD-AEMPKCAND
tr_A0A0K1D8J1_DOLFI      QCFQGS-PFCMCWRKDGSHITDPSI--KIKTCVCHAHRDKELRRSQTGMVGNFIPSCQES
sp_P84032_PHONI          QCYEDS-KFCVCYDKKGHAASPIST--KVKECGCYLKQKERK---DSGRESAIIPQCEED
tr_A0A2P6KRP6_NEPCL      QCYKDS-KFCVCYDKKGHAASPISS--KIKECGCYLRRKEKL---DRNIDDAYIPQCSED
tr_J7FXP5_LATHE          QCFEGS-PFCMCWRPDGSHITDPSL--KLKTCSCIAHRDRV---SQTHLIGNYKPQCEQD
tr_A0A2L2Z2A8_PARTE      QCFEGS-KFCMCWRPDGSHIVDPSL--NVKSCVCHVHNDRELQKSSKVMVGNCIPQFNDD
tr_B5M6G6_HAPSC          QCYPGT-TACMCVAADGSPLTLPGF-VNVTACDCFVEYYKMW---LKDEDSPVLPHCADD
tr_A0A1D0C023_HADIN      QCFKDS-KFCACWDKTGQPMTEPKQ--GIKACECIVQRE-TVQK-IRGLIGAFTPQCEED
tr_A0A224X890_MEGGE      QCHGGT-RYCQCWDKEGHPITAPSM--NVKACECHREKKVAEDK-SKGMVGAFIPTCEDN
tr_A0A1W7R943_HADSP      QCKKNG-VDCQCWRTDGTPINDFSP--NLKACSCIRSKDNA---NRPHLIGNYKPQCEAD
tr_T1DEL1_LYCBU          QCHSEG-RFCSCWRKDGTPITQPST--KIKSCECHRAKDDKLQT-HKGAIGAYIPQCTED
tr_A0A1E1WVQ1_TITOB      QCHNEG-KFCSCWRKDGTPITQPST--KIKSCSCHRDRDDKLKT-SKGLIGAFLPQCGED
                         **       * *    *              * *                    *    .

TT1LP_1_CUPSA            GSYEPLQCSKASGECWCVDRN-GTVLVPPSKD--VHSCNN
TT1LP_2_CUPSA            GSYEPLQCSKASGECWCVDRN-GTVLVPPSKD--VHSCNN
TT1LP_VIRFA              GSYEPLQCSKATGECWCVDRN-GKLLVPPSKD--IHSCSN
tr_A0A0K1D8J1_DOLFI      GYYSRKQCHGSTGYCWCADDN-GNKVSESVRG--TANC--
sp_P84032_PHONI          GKWAKKQLWEFNKSCWCVDEK-GEQVGKIHHDCDSLKCE-
tr_A0A2P6KRP6_NEPCL      GSWVPKQCWDYNDSCWCVDKE-GKQVGDIKAEGKGLNC--
tr_J7FXP5_LATHE          GSYSRTQCHGGMGYCWCVDEH-GQKVNKDLND--C-----
tr_A0A2L2Z2A8_PARTE      GSYPRKQCHGSTGLCWCADET-GK----------------
tr_B5M6G6_HAPSC          GTFKPLQCNKAEGVCWCVDKT-GHVLTKPSKD--TTSC--
tr_A0A1D0C023_HADIN      GKYSKMQCHGSTGHCWCVEQVSGRNVTAPVRG--RLEC--
tr_A0A224X890_MEGGE      GHYSKMQCWGSTGTCWCVDET-GKKTSEPTRD--EVNC--
tr_A0A1W7R943_HADSP      GTYRLTQCWGSVGSCWCVDAE-GRKLPNKHFP--V-DC--
tr_T1DEL1_LYCBU          GRFDKQQCWGSIGMCWCVDIETGRNTTAPSR---NSNC--
tr_A0A1E1WVQ1_TITOB      GKFKKKQCRGSTGQCWCVNQETGEK---------------
                         * :   *       ***.:   *
```

Percent Identity Matrix - created by Clustal2.1

1: TT1LP_1_CUPSA	100.00	99.34	76.82	29.29	27.20	26.43	32.12	26.40	49.66	25.18	25.36	30.22	27.66	25.19
2: TT1LP_2_CUPSA	99.34	100.00	76.16	29.29	27.20	26.43	32.12	26.40	48.98	25.18	25.36	30.22	27.66	25.19
3: TT1LP_VIRFA	76.82	76.16	100.00	31.43	29.60	30.71	35.77	34.40	49.66	28.06	28.99	29.50	27.66	26.72
4: tr_A0A0K1D8J1_DOLFI	29.29	29.29	31.43	100.00	38.40	39.72	63.77	62.79	24.82	42.86	46.04	35.00	45.07	46.97
5: sp_P84032_PHONI	27.20	27.20	29.60	38.40	100.00	70.87	39.67	38.05	24.80	38.71	41.60	28.46	37.90	41.23
6: tr_A0A2P6KRP6_NEPCL	26.43	26.43	30.71	39.72	70.87	100.00	37.96	36.51	25.53	36.23	37.96	30.22	41.43	43.08
7: tr_J7FXP5_LATHE	32.12	32.12	35.77	63.77	39.67	37.96	100.00	63.49	26.81	42.96	39.55	44.60	40.15	46.15
8: tr_A0A2L2Z2A8_PARTE	26.40	26.40	34.40	62.79	38.05	36.51	63.49	100.00	28.57	39.37	44.88	34.92	42.97	43.75
9: tr_B5M6G6_HAPSC	49.66	48.98	49.66	24.82	24.80	25.53	26.81	28.57	100.00	25.00	28.78	25.00	26.76	25.76
10: tr_A0A1D0C023_HADIN	25.18	25.18	28.06	42.86	38.71	36.23	42.96	39.37	25.00	100.00	48.57	37.23	45.07	46.97
11: tr_A0A224X890_MEGGE	25.36	25.36	28.99	46.04	41.60	37.96	39.55	44.88	28.78	48.57	100.00	38.24	51.43	46.15
12: tr_A0A1W7R943_HADSP	30.22	30.22	29.50	35.00	28.46	30.22	44.60	34.92	25.00	37.23	38.24	100.00	42.45	42.31
13: tr_T1DEL1_LYCBU	27.66	27.66	27.66	45.07	37.90	41.43	40.15	42.97	26.76	45.07	51.43	42.45	100.00	72.59

```
14: tr_A0A1E1WVQ1_TITOB  25.19  25.19  26.72  46.97  41.23  43.08  46.15  43.75  25.76  46.97  46.15  42.31  72.59 100.00
```

Proteomic coverage trypsin

>TT1LP_1_S1_CUPSA thyroglobulin type-1-like protein isoform 1

MKAFIVVILA CVATT**SFCWE YPGYPGVDCP TAREKMLEQK DVQWMIPECR ADRTFVDLQC YEKYPDVCMC VAPDGSPLTL PGFGVDPVTC VCFMAQYNIF VHDPEAEMPS CAKDGSYEPL QCSKASGECW CVDRNGTVLV PPSKDVHSCN N**

>TT1LP_2_S1_CUPSA thyroglobulin type-1-like protein isoform 2

MKAFIVVILA CVATTSFC**WE YPGYPGVDCP MAREKMLEQK DVQWMIPECR ADRTFVDLQC YEKYPDVCMC VAPDGSPLTL PGFGVDPVTC VCFMAQYNIF VHDPEAEMPS CAKDGSYEPL QCSKASGECW CVDRNGTVLV PPSKDVHSCN N**

Proteomic coverage chymotrypsin

>TT1LP_1_S1_CUPSA thyroglobulin type-1-like protein isoform 1

MKAFIVVILA CVATTSFCWE Y**PGYPGVDCP TAREKMLEQK DVQWMIPECR ADRTFVDLQC YEKYPDVCMC VAPDGSPLTL PGFGVDPVTC VCFM**AQY**NIF VHDPEAEMPS CAKDGSYEPL QCSKASGECW CVDRNGTVLV PPSKDVHSCN N**

>TT1LP_2_S1_CUPSA thyroglobulin type-1-like protein isoform 2

MKAFIVVILA CVATTSFCWE YPGYPGVDCP **MAREKMLEQK DVQWMIPECR ADRTFVDLQC YEKYPDVCMC VAPDGSPLTL PGFGVDPVTC VCFM**AQY**NIF VHDPEAEMPS CAKDGSYEPL QCSKASGECW CVDRNGTVLV PPSKDVHSCN N**

Supplementary Fig S1.16 Multiple sequence alignment of TT1LP from the venom glands of the spider *C. salei* (TT1LP_1_S1/S2_CUPSA [MH766642/MH766643]; TT1LP_2_S1/S2_CUPSA [MH766644/MH766645]), *V. fasciatus* (TT1LP_VIRFA), *Dolomedes fimbriatus* (tr_A0A0K1D8J1_DOLFI), *P. nigriventer* (sp_P84032_PHONI), *Nephila clavipes* (tr_A0A2P6KRP6_NEPCL), *Latrodectus hesperus* (tr_J7FXP5_LATHE), *P. tepidariorum* (tr_A0A2L2Z2A8_PARTE), *Haplopelma schmidti* (tr_B5M6G6_HAPSC), *Hadronyche infensa* (tr_A0A1D0C023_HADIN), and the scorpions *Megacormus gertschi* (tr_A0A224X890_MEGGE), *Hadrurus spadix* (tr_A0A1W7R943_HADSP), *Lychas buchari* (tr_T1DEL1_LYCBU), and *Tityus obscurus* (tr_A0A1E1WVQ1_TITOB). Signal peptides are marked in black characters, cysteines are marked in white characters with a black box, positively charged amino acids in pink, negatively charged amino acids in blue, hydrophobic amino acids in red and polar amino acids in green. The two identified thyroglobulin type-1like domains are highlighted in yellow for *C. salei*.
